# The prediction of response to treatment using power Doppler in rheumatoid arthritis: a systematic review

**DOI:** 10.1093/rap/rkaf082

**Published:** 2025-07-12

**Authors:** Ummugulsum Gazel, Alan Zhou, Nicholas Jinhyung Kim, Gizem Ayan, Dilek Solmaz, Servet Akar, Sibel Zehra Aydin

**Affiliations:** Faculty of Medicine, Rheumatology, Ottawa Hospital Research Institute, University of Ottawa, Ottawa, ON, Canada; Faculty of Medicine, Internal Medicine, University of Ottawa, Ottawa, ON, Canada; Faculty of Medicine, Internal Medicine, University of Ottawa, Ottawa, ON, Canada; Faculty of Medicine, Rheumatology, Hacettepe University, Ankara, Turkey; Rheumatology, Izmir Katip Celebi Research and Education Hospital, Izmir, Turkey; Rheumatology, Izmir Katip Celebi Research and Education Hospital, Izmir, Turkey; Faculty of Medicine, Rheumatology, Ottawa Hospital Research Institute, University of Ottawa, Ottawa, ON, Canada

**Keywords:** rheumatoid arthritis, ultrasound, power Doppler, treatment, remission

## Abstract

**Objectives:**

There is no consensus on how musculoskeletal ultrasound (US), especially power Doppler (PD) positivity, should inform treatment decisions in RA. We aimed to summarize the literature on whether PD positivity can predict response to intensification of RA therapies in patients with moderate to high clinical disease activity.

**Methods:**

A systematic literature review was performed using a predefined PICO strategy. The titles and abstracts, and subsequently full texts, were independently screened and reviewed by two reviewers and any disagreement was resolved by a third investigator. Studies that investigated the predictive value of PD were included.

**Results:**

Among 2580 abstracts/titles, 13 studies were included. Studies were heterogeneous regarding the inclusion criteria, baseline and new treatments, scanned joints and follow-up duration. In eight studies, patients with higher baseline PD activity had a better response to treatment, mostly with higher reductions in clinical indices. In contrast, two studies found that the probability of achieving clinical remission decreased as the baseline PD score increased. There was no association between baseline PD and the achievement of clinical remission at the follow-up in the remaining three studies.

**Conclusion:**

The baseline Doppler severity may suggest better improvement with higher reductions in composite scores, but this may not be enough to predict a remission state. How US can be used to predict response in RA management requires a well-designed study that will need to be shaped by the existing observations, most importantly identifying the outcome measure that will also be important for daily practice.

Key messages

- Controversies exist regarding the value of ultrasound in predicting response to RA treatment.
- Higher baseline Doppler severity may predict a higher reduction in clinical indices with treatment.

## Introduction

Clinical outcomes for RA have drastically improved in the last several decades due to new therapeutic agents and treatment strategies. The current treatment paradigm utilizes composite clinical indices such as the DAS or Clinical Disease Activity Index (CDAI) to monitor clinical activity and guide therapeutic decisions, ultimately targeting a state of clinical remission [1, 2]. However, these clinical indices can be affected by a multitude of factors other than RA disease activity, including pain, fatigue and physical disability from concomitant non-inflammatory pathologies such as fibromyalgia or osteoarthritis [3]. A reliance on these indices in clinical practice can result in overtreatment and exposure to undue medication side effects in the absence of objective signs of inflammation.

Musculoskeletal ultrasound (US) is a technology that has been shown to improve the accuracy of physical examination [4, 5]. US is often employed at the bedside to identify objective inflammation and is particularly helpful when patient symptoms do not correlate with findings on physical examination [6]. Two randomized controlled trials (RCTs) that focused on treating subclinical disease failed to demonstrate improved outcomes with US-driven treat-to-target approaches [7, 8]. However, there are no RCTs on the role of US in informing treatment decisions for RA therapy escalation when there is a discrepancy between the patient and the physician, with the patient scoring higher, although this applies to one of the most common uses of musculoskeletal US in clinical practice [9]. The objective of our study was to summarize the existing literature to determine whether power Doppler US (PDUS) activity can predict response to the intensification of RA therapies in patients with moderate to high clinical disease activity.

## Methods

### Search and selection strategy

A systematic literature review was performed using a predefined PICO (population, intervention, comparator and outcome) strategy on the MEDLINE, Embase and Cochrane Central Register databases by an experienced librarian at the University of Ottawa. Details of the search strategy are provided in Supplementary Table S1, available at *Rheumatology Advances in Practice* online. Since this is a systematic literature review that does not involve human subjects, ethics approval was not required. The study protocol was registered with the International Prospective Register of Systematic Reviews (PROSPERO: CRD42021258344). The literature search was performed from January 2000 to November 2023. To be eligible for inclusion, studies had to meet the following criteria: RCTs, observational studies including case–control studies, cohort studies, nested case–control studies and cross-sectional studies with assessment of the prediction of PD for treatment response. Our search identified 2580 abstracts. We excluded articles with incomplete data, reporting bias, duplication, case reports, review articles, consensus reports and languages other than English. An overview of our literature search and screening results are outlined in Supplementary Fig. S1 and Supplementary Table S1, available at *Rheumatology Advances in Practice* online.

### Data extraction

The titles and abstracts were independently screened by two of three reviewers (U.G.G., A.Z., N.K.). All abstracts with a discrepancy were carried to a full-text review. The full texts were reviewed independently by the same investigators. A third investigator (S.Z.A.) resolved any disagreement at this stage. In addition, the references of all included articles were manually scanned. Articles that did not fulfil the inclusion criteria were identified and the reason for exclusion was documented. The following data were extracted: publication year, study design, sample size, sex distribution, baseline and new treatment, change in disease activity parameter and results for the prediction.

### Quality assessment

The National Heart, Lung, and Blood Institute of the National Institutes of Health tool for quality assessment of observational cohort and cross-sectional studies was used to assess the quality of all included studies [10]. The studies were divided into three groups: fair, poor and good.

## Results

We identified 2580 abstracts and titles. After the duplicates were removed, 2515 were screened. Among 149 studies eligible for full-text review, 136 studies were excluded. Reasons for exclusion during the full-text review were eight studies for being only study protocols, 2 in the wrong language, 46 poster presentations, 2 wrong study designs, 1 included <10 patients and 77 for wrong study outcomes (details provided in Supplementary Fig. S1 and Supplementary Table S2, available at *Rheumatology Advances in Practice* online). After the full-text review, 13 studies were found to be eligible for inclusion.

### Study designs and patient profiles

All the studies’ designs were prospective observational, except Kawashiri *et al.*’s study, which was a retrospective data collection [11]. The sample size of the studies varied from 10 to 141. The mean age ranged from 46 to 59 years and sex dominance was female in all. At recruitment, the patients were treatment naïve in one study [12], biologic DMARD (bDMARD) naïve in five studies [13–17] and on bDMARDs or conventional synthetic DMARDs in five studies [11, 18–21]. Baseline treatment was not reported in three studies [22, 23]. In seven studies, patients had to have active disease at baseline according to the 28-joint DAS (DAS28) with CRP, ESR or CDAI in various cut-offs before the new therapy [12, 14, 16, 18–20, 23]. Also, in Razmjou *et al.*’s study [18], having a total PDUS ≥10, and in Ranganath *et al.*’s study [16], having a PDUS >1 in at least one joint was one of the inclusion criterion. In four studies, a new treatment was either biologic or conventional DMARDs [12, 13, 20, 23], while in the rest of the studies, only patients who started a biologic treatment were included. The duration of follow-up ranged from 3 to 12 months.

### US scanning and scoring

There was heterogeneity in terms of the joints that were assessed by the US. Two studies included only the wrist joint from the dorsal view and one included only the MCP joints [14, 17, 22]. The other studies assessed multiple joints by US. Most of the studies used a semiquantitative PD score of 0–3 per joint, resulting in an overall PD score per patient. Overall PD scores changed based on the number of joints scanned by US, with the upper limit ranging from 39 to 102. Three of thirteen studies used a cut-off for the PD score for the analysis [12, 16, 17]. Table 1 and Supplementary Table S3, available at *Rheumatology Advances in Practice* online, display detailed information regarding the study characteristics.

**Table 1.** Study characteristics.

Author	Sample size^c^, *n*	Age, years, mean (s.d.)	Sex(male/female), *n*/*n*	Baseline treatment at study entry		New treatment	Follow-up duration	Joints assessed with US, *n*	US Doppler parameter	PD score range	PD cut-off used for analysis
				csDMARD	bDMARD						
Gazel *et al.*, 2023 [20]^f^	20	57 (16.9)	9/10	NR	NR	Any treatment modification	3–6 months	36	Semiquantitative PD scoring of 0–3	0–3	>1 PD positivity
Ceccarelli *et al.*, 2022 [21]^f^	102	59.2 (17.7)	15/87	51 (50%)	NR	Baricitinib or tofacitinib	Median 6 months (IQR 3)	22	Semiquantitative PD scoring of 0–3	0–66	NA
Morris *et al.*, 2021 [19]^f^	44	51.9 (15.2)	5/49	100%	0%	Tocilizumab 4 mg/kg every 4 weeks	24 weeks	34	Semiquantitative PD scoring of 0–3	0–3	NA
Razmjou *et al.*, 2020 [18]^f^	24	52 (9.9)	3/22	72%	32%	Tofacitinib	12 weeks	34	Semiquantitative PD scoring of 0–3	0–102	NA
Sapundzhieva *et al.*, 2018 [23]^f^	141	58.90 (11.04)	30/111	NR	NR	sDMARDs 55% or bDMARDs 45%	12 months	7	Semiquantitative PD scoring of 0–3 (joints and tendons, per US-7 score)^h,^^b^	0–39	NA
Kawashiri *et al.*, 2017 [11]^f^	39	47.1 (12.4)	8/31	84%	30.8%	bDMARDs	6 months	22	Semiquantitative PD scoring of 0–3	0–66	NA
Christensen *et al.*, 2016 [13]^d^	98	csDMARD: 55.8 (16.2) bDMARD: 54.0 (15.7)	22/76	csDMARD initiation group 76%, bDMARD initiation group 51%	csDMARD initiation group 0%, bDMARD initiation group 0%	csDMARD 48%, bDMARD 52%	4 months	24 projections in 11 joints^a^	Semiquantitative PD scoring of 0–3^g^	0–72	NA
Horton *et al.*, 2016 [12]^d^	105	59 (13)	26/79	0%	0%	csDMARD 91%, bDMARD 5%, steroids 4%	12 months	26	Semiquantitative PD scoring of 0–3	0–78	Three definitions were used: (1) absence of PDA activity in 26 joints (total PDA score = 0); (2) absent/minimal PDA activity in 26 joints ≤1; (3) absent/minimal PDA activity in 26 joints (total PDA score ≤1, excluding PDA grade 1 in the wrists and/or first MTPs)
Hull *et al.*, 2016 [14]^e^	25	59 (13)	7/18	100% (treated with at least two DMARDs)	0%	Anti-TNF-α treatment	12 weeks	10 (MCP joints only)	Synovial vascularity score (trans-PDA)^a^	NR	NA
Inanc *et al.*, 2016 [15]^e^	39	Responders: 46.4 (11.6) Non-responders: 47.1 (12.4)	9/30	NR	0%	Anti-TNF-α treatment	3 months	28	Semiquantitative PD scoring of 0–3	0–84	NA
Ranganath *et al.*, 2015 [16]^e^	19	NR	NR	NR	0%	Abatacept	12 months	7	Semiquantitative PD scoring of 0–3	NR	PDUS ≥ 5 and PDUS ≤5 (prediction analysis is not based on this)
Ellegaard *et al.*, 2014 [22]^e^	46	57.9 (13.9)	9/37	NR	NR	Anti-TNF-α treatment	12 months	1 (dorsal wrist only)	Semiquantitative PD scoring of 0–3 and quantitative colour fraction	PDUS 0–3, CF 0–1	NA
Ellegaard *et al.*, 2011 [17]^e^	109	57.9 (13.9)	31/78	MTX 49.5%;SSZ 10.1%	0%	Anti-TNF-α treatment	12 months	1 (wrist only)	Colour fraction (CF) and √CF^b^	NR	√CF > 0.23

a The power Doppler area (PDA) is a count of the number of pixels with PDUS signal within the defined region of interest. Synovial vascularity score (trans-PDA) is the sum of all joints.

b The CF is the number of colour pixels divided by the total number of pixels in a region of interest.

c Final sample size that is included in the analysis is displayed.

d 1987 ACR and/or 2010 ACR/EULAR criteria.

e 1987 ACR criteria.

f 2010 ACR/EULAR criteria.

g MCP 2–4 (dorsal projections); wrist central, radial and ulnar projections; m. extensor carpi ulnaris tendon; elbow (posterior projection); knee suprapatellar, lateral and medial projections; ankle central, medial and lateral projections; tarsometatarsal central, medial and lateral projections; MTP 24; m. tibialis posterior tendon; and m. peroneus longus et brevis tendons.

h Seven joints of the clinically dominant hand/foot, affected more by swelling or tenderness, using the German US-7 score wrist, second and third MCP and PIP, second and fifth MTP joints, Palmar scan was used to assess MCP2 and MCP3 for synovitis and tenosynovitis and dorsal scan for paratenonitis.

### Definition of response, primary outcomes and analysis

Two different outcome measures were used to define improvement of disease activity: six studies used improvement in the DAS28 and/or CDAI scores [13, 16, 18, 19, 21, 22] and one used the change in tender joint count (TJC) and swollen joint count (SJC) [20]. Six studies used achieving remission after treatment [11, 12, 14, 15, 17, 23]. The mean/median disease activity results for the baseline and follow-up, as well as the difference, are given in Table 2. Different analyses were used in the studies, including correlation, regression and effect size.

**Table 2.** Outcome results of the studies.

Author	Analysis	Outcome	Results	Conclusion
Studies that found better response to treatment with higher PD score at baseline				
Gazel *et al.*, 2022 [20]	Percentage of change in clinical activity indices	Mean change in TJC and SJC^a,c^	ΔTJC: PD-positive patients: 5.1, PD-negative patients: 1.1 ΔSJC: PD-positive patients : 3.5, PD-negative patients : 1.1 ΔVAS: PD-positive patients: 3.1, PD-negative patients: 0.7	Simple definition of the presence of moderate PD positivity in one joint may predict favourable clinical responses to treatment alterations
Ceccarelli *et al.*, 2022 [21]	Significance of change in disease activity indices over time; multivariate regression analysis	Change in DAS28-CRP in weeks 12, 24 and 48	Tenosynovitis US score independently associated with changes in DAS28-CRP from baseline to 4 and 12 weeks (*P* *=* 0.02 and *P* *=* 0.001, respectively). Similarly, baseline PD US score was independently associated with DAS28-CRP changes at only 12 weeks follow-up (*P* *=* 0.04). Patients in remission or low-disease activity during the follow-up had higher baseline tenosynovitis score at baseline [median 3 (IQR 4)] compared with those in moderate or high disease activity [median 0 (IQR 2)] (*P* *=* 0.01)	PD and tenosynovitis scores could play a predictive role in response to treatment with JAK inhibitors in RA patients
Morris *et al.*, 2021 [19]	Correlation analysis of baseline PD score with DAS28‐ESR and CDAI	Change in disease activity measures (CDAI and DAS28-ESR)	Baseline PDUS scores and 12-week change is significantly correlated with changes in CDAI from 12 to 24 weeks (*r* = 0.42, *P* *<* 0.01)	Baseline US34-PDUS and its early changes after initiating biologic therapy may be a useful predictor for IV-TCZ response in RA patients
Razmjou *et al.*, 2020 [18]	Pearson correlations for associations between baseline US and changes in CDAI or DAS28; multiple linear regression models for the outcomes of change in clinical indices	Change in CDAI and DAS28 scores	Baseline PDUS scores correlated significantly with changes in CDAI and DAS28 (range: *r* = 0.53–0.58; *P* *<* 0.01). Multiple linear regression demonstrated a positive independent association between baseline PDUS and change in DAS28 from baseline to week 6 (*P* *=* 0.01), with adjustment for baseline DAS28, similar to the same analysis with CDAI (*P* *=* 0.004. Baseline PDUS showed a trend toward a positive association with changes in CDAI and DAS28 from baseline to week 12 (*P* *=* 0.07 and *P* *=* 0.05, respectively)	Higher baseline PDUS values are associated with larger CDAI and DAS28 responses at 6 weeks, with a trend observed at 12 weeks
Christensen *et al.*, 2016 [13]	Multiple regression analysis	Change in DAS28 score	Unadjusted model: USD score β 0.057 (95% CI 0.024, 0.090), *P* *≤* 0.001; TS β −0.152 (95% CI 0.649, 0.345), *P* *=* 0.55. Adjusted model: USD score β 0.032 (95% CI 0.001, 0.063), *P* *=* 0.04)	USD score was an independent predictor of the change in DAS28, but TS of pain was not. Patients who had more inflammation (assessed by US) at baseline obtained significantly greater DAS28 improvements at the follow-up
Hull *et al.*, 2016 [14]	Wilcoxon matched pairs test to compare baseline trans-PDA score with follow-up	Number of patients who achieved improvement in the DAS28 score >1.2 from baseline to 12 weeks	Baseline Doppler score is numerically higher in responders than non-responders: 3213 (115–6772)^b^ *vs* 25 (0–1079)^b^	The quantitative measures of synovial thickening and synovial vascularity were both able to discriminate between EULAR good responders and non-responders to anti-TNF treatment
Ranganath *et al.*, 2015 [16]	Correlation between the baseline PDUS and change in DAS28; multivariate linear regression analysis	Change in DAS28-ESR score	Baseline PDUS was significantly correlated with the change in DAS28-ESR at 12 months (DAS28-ESR = 0.65, *P* < 0.01). In multivariate regression analysis: baseline PDUS was almost significantly associated with the change in DAS28-ESR (*P* = 0.06) after accounting for baseline DAS28-ESR (*P* = 0.52)	Patients with high baseline PDUS had a more robust response to therapy
Ellegaard *et al.*, 2011 [17]	Effect size	Percent of completers *vs* drop-outs (due to lack of efficacy)	Mean CF between completers 0.270 (s.d. 0.180) *vs* drop-outs 0.179 (s.d. 0.151), *P* = 0.020, effect size = 0.51 Mean √CF between completers 0.484 (s.d. 0.188) *vs* drop-outs 0.371 (s.d. 0.206), *P* = 0.024, effect size = 0.57	Higher US Doppler measurement using CF obtained at baseline was the only outcome measure that could significantly predict which patients would remain on anti-TNF therapy
Studies that found less response to treatment with higher PD score at baseline				
Sapundzhieva *et al.*, 2018 [23]	Binary logistic regression, Spearman rho correlation analysis	Number of patients in DAS28 remission	For sDMARD group: the patients without DAS28 remission had significantly higher mean values for PDUS score at baseline than those with DAS28 remission. Baseline PDUS for DAS28 remission = 8.56 (s.d. 4.62) (median 7), non-remission = 5.76 (s.d. 3.22) (median 5), *P* = 0.003. Prognostic model for DAS28 suggested the PDUS as an independent variable: *P* = 0.038, B: −0.184 Spearman correlation analysis: baseline PDUS and DAS28 remission (*r* = −0.517, *P* < 0.001) For bDMARD group: the patients without DAS28 remission had significantly higher mean scores than those with DAS28 remission. Baseline PDUS for DAS28 remission = 8.75 (s.d. 4.84) (median 7), non-remission = 6.06 (s.d. 3.18) (median 5), *P* = 0.006. Prognostic model for DAS28 suggested the PDUS as an independent variable: *P* = 0.035, B: −0.174 Spearman correlation analysis: baseline PDUS and DAS28 remission (*r* = −0.393, *P* = 0.001)	The probability of achieving DAS28 remission decreases as baseline PDUS score increases for both the csDMARD and bDMARD therapies
Inanc *et al.*, 2016 [15]	Stepwise-multivariable logistic regression	Number of patients in EULAR response (good/moderate)	Baseline PD sum score in responders = 13.0 (s.d. 5.1) *vs* non-responders = 20.6 (s.d. 11.8), *P* = 0.035. In multivariable analysis, only baseline the PD synovitis sum score [OR 0.86 (95% CI 0.75, 0.98), *P* = 0.023] was independently associated with EULAR response to TNF inhibitor at month 3	Baseline PD scores were able to predict responders *vs* non-responders to TNF inhibitors at 3 months in seropositive RA patients, while baseline clinical features were not (DAS28). Non-responder patients have excess inflammatory activity that is detectable with PDUS but not with clinical examination
Studies that found no association between response to treatment and with PD score at baseline				
Kawashiri *et al.*, 2017 [11]	Mann–Whitney U test and Wilcoxon’s signed rank test	Number of patients in clinical remission according to EULAR good response and DAS28-ESR remission	EULAR moderate and good responders PD score = 8 *vs* non-responders PD score = 7, *P* = 0.74; EULAR good responders PD score = 10 *vs* non-responders PD score = 7, *P* = 0.45; DAS28-ESR remission PD score = 9 *vs* non-remission PD score = 7, *P* = 0.33	There were no differences for baseline PD score according to clinical remission at 6 months
Horton *et al.*, 2016 [12]	Univariate and multivariate multiple regression analysis	Number of patients in remission according to DAS28-CRP4v remission	Univariable analysis: baseline total PDA score for DAS28-CRP4v 0.98 (95% CI 0.92, 1.05), *P* = NS; for DAS44-CRP4v 0.97 (95% CI 0.91, 1.04), *P* = NS; for Boolean 0.98 (95% CI 0.89, 1.07)	Baseline PDA did not predict DAS28-CRP4v remission in new-onset RA patients
Ellegaard *et al.*, 2014 [22]	Spearman’s rank order correlation analysis	Change in DAS28 score	NR	The discriminative ability (ability to predict treatment success measured as decrease in DAS28) was poor for both PD scoring systems (data not shown)

a Mean (s.d.).

b Median (IQR).

c Mean (95% CI).

### Prediction with the baseline PD

In eight studies, authors found that patients who had higher baseline PD activity had a better response to treatment. Among these, five studies found that patients who had higher PD scores at baseline had greater reductions in clinical indices compared with patients with lower PD scores [13, 16, 18–21]. Similarly, Hull *et al.* [14] found that synovial vascularity was higher in patients who were good EULAR responders at follow-up. Also, in Ellegaard *et al.*’s study [17], patients with higher PDUS scores at baseline remained on anti-TNF therapy longer. In contrast with these findings, two studies found that the probability of achieving clinical remission decreases as the baseline PDUS score increases [15, 23]. In the other three studies, there was no association between baseline PDUS and the achievement of clinical remission at the follow-up after treatment escalation for the disease activity. Table 2 and Supplementary Table S3, available at *Rheumatology Advances in Practice* online, provide details regarding the study results.

## Quality assessment

In 10 studies included in our review, 7 were rated as ‘good’, 4 articles as ‘fair’ and 2 articles as ‘poor’ (Supplementary Table S4, available at *Rheumatology Advances in Practice* online).

## Discussion

The utility of US to modify clinicians’ treatment choices in real life has been demonstrated, but the way it has been utilized has yet to be proven effective [24]. Previous observational studies showed that the presence of PD-positive synovitis might be more helpful than clinical examination in predicting flares in patients who are in clinical remission, suggesting that sonography can help clinicians decide how to manage patients in remission [25–27]. Also, there is evidence that PD positivity may predict radiographic damage progression in RA patients [28, 29]. However, despite the superiority of US over clinical assessment to detect inflammation, how to incorporate that information into daily practice is unclear. In this study, our results showed that there is a controversy regarding studies evaluating the role of baseline PD positivity for treatment response in RA patients. The majority (8/13) of the studies showed that patients with higher baseline PD activity had a better response to treatment, while two studies found the opposite and the other three found no association. Although these controversial findings can suggest an unclear association between baseline Doppler findings and the response, we observed major differences in the studies’ designs, which can also explain the different results achieved. Some differences included the inclusion criterion, treatments and the number of scanned joints, which may all contribute. However, we believe the biggest difference is the primary outcome that was used in these studies. In eight studies that found PD to have a predictive value for better response, six of them identified the outcome as the change in DAS scores [13, 14, 16, 18, 19, 21]. In contrast, within the other five studies that found the opposite or were found to be neutral, in four of them, the outcome was chosen as a disease state or included the disease state in the definition of response (EULAR good or moderate response) [11, 12, 15, 23]. These differences indicate that the baseline Doppler severity may suggest a greater reduction in composite scores, but this may not be enough to predict a remission state.

Based on previous research regarding clinical disease activity scores, patients with high disease activity at baseline appear to have a greater potential for favourable therapeutic responses [30]. This finding is consistent with US assessments; however, a key distinction emerges in that patients with similar clinical disease activity scores may exhibit differing levels of US-detected synovitis. Therefore, US may play a critical role in further stratifying disease activity and guiding management in this regard.

Our findings are limited by the heterogeneity of the studies analysed, preventing us from drawing robust conclusions. Nevertheless, we believe it is essential to highlight this issue as a critical area for future investigation, supported by evidence from the current literature. Our research group is presently conducting an RCT to evaluate the feasibility of a study design aimed at determining whether the integration of US influences treatment outcomes in newly diagnosed RA patients with high disease activity. Additionally, as part of our research agenda, we plan to investigate other objective features in RA, such as functional MRI, to gain a deeper understanding of pain mechanisms. Given that pain may arise from multiple sources, it can confound clinical disease activity assessments, underscoring the need for more precise evaluation tools [31].

In conclusion, the role of baseline Doppler activity in predicting remission in RA is still controversial. The heterogeneity between studies is likely to affect the results of the existing literature and make it challenging to draw conclusions. How US can be used to predict response in RA management requires well-designed studies that will need to be shaped on the existing observations, such as determining the right study outcome (response *vs* disease state). The wide range of patient profiles, based on disease durations, disease activities and/or baseline and new therapies, may require more than one study design to understand the predictive value of Doppler positivity in different scenarios. With the cumulative data of studies exploring this question, we will be able to understand the best way to incorporate US in the management algorithms in RA.

## Supplementary Material

rkaf082_Supplementary_Data

## Data Availability

The data underlying this article will be shared upon reasonable request to the corresponding author.

## References

[1] Smolen JS , Aletaha D, Bijlsma JWJ et al Treating rheumatoid arthritis to target: recommendations of an international task force. Ann Rheum Dis 2010;69:631–7. 20215140 10.1136/ard.2009.123919 PMC3015099

[2] Aletaha D , Smolen J. The Simplified Disease Activity Index (SDAI) and the Clinical Disease Activity Index (CDAI): a review of their usefulness and validity in rheumatoid arthritis. Clin Exp Rheumatol 2005;23(5 Suppl 39):S100–8. 16273793

[3] Vermeer M , Kuper HH, van der Bijl AE et al The provisional ACR/EULAR definition of remission in RA: a comment on the patient global assessment criterion. Rheumatology (Oxford) 2012;51:1076–80. 22302059 10.1093/rheumatology/ker425

[4] Wakefield RJ , Green MJ, Marzo-Ortega H et al Should oligoarthritis be reclassified? Ultrasound reveals a high prevalence of subclinical disease. Ann Rheum Dis 2004;63:382–5. 15020331 10.1136/ard.2003.007062 PMC1754934

[5] Saleem B , Brown AK, Keen H et al Should imaging be a component of rheumatoid arthritis remission criteria? A comparison between traditional and modified composite remission scores and imaging assessments. Ann Rheum Dis 2011;70:792–8. 21242236 10.1136/ard.2010.134445

[6] Díaz-Torné C , Moragues C, Toniolo E et al Impact of ultrasonography on treatment decision in rheumatoid arthritis: the IMPULSAR study. Rheumatol Int 2017;37:891–6. 28258474 10.1007/s00296-017-3689-2

[7] Dale J , Stirling A, Zhang R et al Targeting ultrasound remission in early rheumatoid arthritis: the results of the TaSER study, a randomised clinical trial. Ann Rheum Dis 2016;75:1043–50. 27026689 10.1136/annrheumdis-2015-208941

[8] Haavardsholm EA , Aga A-B, Olsen IC et al Ultrasound in management of rheumatoid arthritis: ARCTIC randomised controlled strategy trial. BMJ 2016;354:i4205. 27530741 10.1136/bmj.i4205 PMC4986519

[9] Di Matteo A , Mankia K, Azukizawa M, Wakefield RJ. The role of musculoskeletal ultrasound in the rheumatoid arthritis continuum. Curr Rheumatol Rep 2020;22:41. 32562012 10.1007/s11926-020-00911-w PMC7305070

[10] National Heart, Lung, and Blood Institute. Study quality assessment tools. https://www-nhlbi-nih-gov.proxy.bib.uottawa.ca/health-topics/study-quality-assessment-tools (1 August 2025, date last accessed).

[11] Kawashiri S-Y , Nishino A, Shimizu T et al Ultrasound disease activity of bilateral wrist and finger joints at three months reflects the clinical response at six months of patients with rheumatoid arthritis treated with biologic disease-modifying anti-rheumatic drugs. Mod Rheumatol 2017;27:252–6. 27585858 10.1080/14397595.2016.1221874

[12] Horton SC , Tan AL, Freeston JE et al Discordance between the predictors of clinical and imaging remission in patients with early rheumatoid arthritis in clinical practice: implications for the use of ultrasound within a treatment-to-target strategy. Rheumatology (Oxford) 2016;55:1177–87. 26998766 10.1093/rheumatology/kew037

[13] Christensen AW , Rifbjerg-Madsen S, Christensen R et al Ultrasound Doppler but not temporal summation of pain predicts DAS28 response in rheumatoid arthritis: a prospective cohort study. Rheumatology (Oxford) 2016;55:1091–8. 26983452 10.1093/rheumatology/kew034

[14] Hull DN , Cooksley H, Chokshi S et al Increase in circulating Th17 cells during anti-TNF therapy is associated with ultrasonographic improvement of synovitis in rheumatoid arthritis. Arthritis Res Ther 2016;18:303. 28010726 10.1186/s13075-016-1197-5 PMC5180397

[15] Inanc N , Ozen G, Direskeneli H. Predictive value of ultrasonographic assessment of disease activity in response to tumor necrosis factor-alpha inhibitor treatment in rheumatoid arthritis: a prospective cohort study. Clin Exp Rheumatol 2016;34:156. 26575409

[16] Ranganath VK , Ben-Artzi A, Duffy E et al Elevated baseline power Doppler discriminates an RA subgroup highly responsive to therapy. Rheumatology (Oxford) 2015;54:2285–6. 26316582 10.1093/rheumatology/kev301 PMC4938871

[17] Ellegaard K , Christensen R, Torp-Pedersen S et al Ultrasound Doppler measurements predict success of treatment with anti-TNF-α; drug in patients with rheumatoid arthritis: a prospective cohort study. Rheumatology (Oxford) 2011;50:506–12. 21071479 10.1093/rheumatology/keq336

[18] Razmjou AA , Brook J, Elashoff D et al Ultrasound and multi-biomarker disease activity score for assessing and predicting clinical response to tofacitinib treatment in patients with rheumatoid arthritis. BMC Rheumatol 2020;4:55. 33089069 10.1186/s41927-020-00153-4 PMC7569763

[19] Morris NT , Brook J, Ben-Artzi A et al Doppler ultrasound impacts response to intravenous tocilizumab in rheumatoid arthritis patients. Clin Rheumatol 2021;40:5055–65. 34269927 10.1007/s10067-021-05857-7

[20] Gazel U , Ayan G, Solmaz D et al Prediction of response to treatment using Doppler signal positivity measured by ultrasound in rheumatoid arthritis: a proof-of-concept study. Eur J Rheumatol 2023;10:118–9. 37594393 10.5152/eurjrheum.2023.23005 PMC10544597

[21] Ceccarelli F , Spinelli FR, Garufi C et al The role of musculoskeletal ultrasound in predicting the response to JAK inhibitors: results from a monocentric cohort. Clin Exp Rheumatol 2022;40:921–7. 34251313 10.55563/clinexprheumatol/totvyv

[22] Ellegaard K , Terslev L, Christensen R et al Comparison of discrimination and prognostic value of two US Doppler scoring systems in rheumatoid arthritis patients: a prospective cohort study. Clin Exp Rheumatol 2014;32:495–500. 24960526

[23] Sapundzhieva T , Karalilova R, Batalov A. Musculoskeletal ultrasound for predicting remission in patients with rheumatoid arthritis: results from a 1-year prospective study. Rheumatol Int 2018;38:1891–9. 30121699 10.1007/s00296-018-4131-0

[24] Dale J , Purves D, McConnachie A, McInnes I, Porter D. Tightening up? Impact of musculoskeletal ultrasound disease activity assessment on early rheumatoid arthritis patients treated using a treat to target strategy. Arthritis Care Res (Hoboken) 2014;66:19–26. 24376248 10.1002/acr.22218

[25] Saleem B , Brown AK, Quinn M et al Can flare be predicted in DMARD treated RA patients in remission, and is it important? A cohort study. Ann Rheum Dis 2012;71:1316–21. 22294638 10.1136/annrheumdis-2011-200548

[26] Scire CA , Montecucco C, Codullo V et al Ultrasonographic evaluation of joint involvement in early rheumatoid arthritis in clinical remission: power Doppler signal predicts short-term relapse. Rheumatology (Oxford) 2009;48:1092–7. 19561156 10.1093/rheumatology/kep171

[27] Janta I , Valor L, De la Torre I et al Ultrasound-detected activity in rheumatoid arthritis on methotrexate therapy: which joints and tendons should be assessed to predict unstable remission? Rheumatol Int 2016;36:387–96. 26712373 10.1007/s00296-015-3409-8

[28] Ikeda K , Nakagomi D, Sanayama Y et al Correlation of radiographic progression with the cumulative activity of synovitis estimated by power Doppler ultrasound in rheumatoid arthritis: difference between patients treated with methotrexate and those treated with biological agents. J Rheumatol 2013;40:1967–76. 24187096 10.3899/jrheum.130556

[29] Funck-Brentano T , Gandjbakhch F, Etchepare F et al Prediction of radiographic damage in early arthritis by sonographic erosions and power Doppler signal: a longitudinal observational study. Arthritis Care Res (Hoboken) 2013;65:896–902. 23212992 10.1002/acr.21912

[30] Kleinert S , Tony H-P, Krause A et al Impact of patient and disease characteristics on therapeutic success during adalimumab treatment of patients with rheumatoid arthritis: data from a German noninterventional observational study. Rheumatol Int 2012;32:2759–67. 21822659 10.1007/s00296-011-2033-5 PMC3429775

[31] Gazel UNK , Ayan B, Ayan G et al Pain mechanisms in psoriatic arthritis: differentiating inflammation related pain in enthesitis using ultrasound, in comparison to functional MRI [abstract]. Arthritis Rheumatol 2023;75(Suppl 9):abstract 1388.

