# Supplementary material for: The prediction of response to treatment using power Doppler in rheumatoid arthritis: a systematic review
Source: Rheumatol Adv Pract. 2025 Jul 12;9(3):rkaf082. doi: 10.1093/rap/rkaf082 (PMC12360839; doi:10.1093/rap/rkaf082)
Supplement: rkaf082_Supplementary_Data [file rkaf082_supplementary_data.docx]

**Supplementary Table S1. Search strategy**

| Ovid MEDLINE(R) ALL <1946 to October 31, 2023>  1 exp Arthritis, Rheumatoid/ 126592  2 ((rheumatoid or rheumatic) adj3 arthritis).tw,kf. 128896  3 ((rheumatoid or rheumatic) adj (diseas* or condition* or nodule*)).tw,kf. 23157  4 Caplan Syndrome*.tw,kf. 32  5 Felty Syndrome*.tw,kf. 108  6 Rheumatoid Vasculitis.tw,kf. 416  7 Sjogren* Syndrome*.tw,kf. 18225  8 Still* Disease*.tw,kf. 2973  9 1 or 2 or 3 or 4 or 5 or 6 or 7 or 8 187525  10 doppler*.mp. 150880  11 9 and 10 1392  12 Leflunomide.mp. 2879  13 Hydroxychloroquine.mp. 10566  14 Methotrexate.mp. 61045  15 Sulfasalazine.mp. 6478  16 Tumor Necrosis Factor-alpha/ai [Antagonists & Inhibitors] 16384  17 (adalimumab or certolizumab pegol or etanercept or infliximab).mp. 29018  18 Antibodies, Monoclonal/ 201357  19 Golimumab.mp. 1674  20 (bDMARD* or dmard*).tw,kf. 7481  21 Disease-Modifying Antirheumatic Drug*.tw,kf. 5553  22 Abatacept.mp. 4530  23 Rituximab.mp. 31343  24 Antibodies, Monoclonal, Humanized/ 53696  25 Tocilizumab.mp. 6755  26 Sarilumab.mp. 362  27 Anakinra.mp. 2692  28 exp Antirheumatic Agents/ 470709  29 Tofacitinib.mp. 3017  30 Baricitinib.mp. 1403  31 exp Glucocorticoids/ 208141  32 Glucocorticoid*.tw,kf. 80792  33 tnf alfa inhibit*.tw,kf. 17  34 Tumor Necrosis Factor* alpha inhibit*.tw,kf. 1271  35 dt.fs. or therapy.tw,kf. 4269953  36 "Severity of Illness Index"/ 271236  37 (disease activity or remission).tw,kf. 189726  38 "Predictive Value of Tests"/ or "Sensitivity and Specificity"/ 557523  39 (predict* or accurac*).tw,kf. or sensitiv*.tw,kw. 3854656  40 or/12-39 8509961  41 11 and 40 1007  42 exp animals/ not humans/ 5164662  43 41 not 42 1000  44 limit 43 to yr="2000 -Current" 981  45 limit 44 to dt=20210409-20231031 109  Embase Classic+Embase <1947 to 2023 October 31>  1 exp *rheumatoid arthritis/ 159692  2 ((rheumatoid or rheumatic) adj3 arthritis).tw. 197668  3 ((rheumatoid or rheumatic) adj (diseas* or condition* or nodule*)).tw. 40069  4 Caplan Syndrome*.tw. 41  5 Felty Syndrome*.tw. 152  6 Sjogren* Syndrome*.tw. 26566  7 Still* Disease*.tw. 4341  8 or/1-7 279021  9 exp *Doppler flowmetry/ or exp *doppler ultrasonography/ 25019  10 doppler*.tw. 172485  11 9 or 10 175948  12 8 and 11 3165  13 *leflunomide/ 2054  14 Leflunomide.tw. 5203  15 *hydroxychloroquine sulfate/ 226  16 *hydroxychloroquine/ 6956  17 Hydroxychloroquine.tw. 15707  18 *methotrexate/ 55410  19 Methotrexate.tw. 83078  20 *salazosulfapyridine/ 6213  21 Sulfasalazine.tw. 6681  22 exp *tumor necrosis factor inhibitor/ 39802  23 (adalimumab or certolizumab pegol or etanercept or infliximab).tw. 52883  24 *golimumab/ 1790  25 Golimumab.tw. 4805  26 *disease modifying antirheumatic drug/ or exp *antirheumatic agent/ 339670  27 (bDMARD* or dmard*).tw. 21718  28 Disease-Modifying Antirheumatic Drug*.tw. 8588  29 Abatacept.tw. 5827  30 Rituximab.tw. 61649  31 Tocilizumab.tw. 13527  32 Sarilumab.tw. 681  33 Anakinra.tw. 5626  34 Tofacitinib.tw. 5797  35 Baricitinib.tw. 2468  36 exp *glucocorticoid/ 269572  37 Glucocorticoid*.tw. 109101  38 Tumor Necrosis Factor* alpha inhibit*.tw. 1544  39 (tnf adj2 inhibit*).tw. 19407  40 *drug therapy/ or *disease activity/ or *remission/ 273736  41 (disease activity or remission).tw. 338798  42 (sensitivity or predict*).tw. 3854063  43 or/13-42 4984319  44 12 and 43 2169  45 (exp animal/ or nonhuman/) not exp human/ 7968605  46 44 not 45 2156  47 limit 46 to yr="2000 -Current" 2144  48 limit 47 to dc=20210409-20231031 314  EBM Reviews - Cochrane Central Register of Controlled Trials <September 2023>  1 exp Arthritis, Rheumatoid/ 7294  2 ((rheumatoid or rheumatic) adj3 arthritis).tw,kw. 17642  3 ((rheumatoid or rheumatic) adj (diseas* or condition* or nodule*)).tw,kw. 2379  4 Caplan Syndrome*.tw,kw. 0  5 Felty Syndrome*.tw,kw.4  6 Rheumatoid Vasculitis.tw,kw. 7  7 Sjogren* Syndrome*.tw,kw. 907  8 Still* Disease*.tw,kw. 77  9 1 or 2 or 3 or 4 or 5 or 6 or 7 or 8 20579  10 doppler*.mp. 13179  11 9 and 10 241  12 Leflunomide.mp. 723  13 Hydroxychloroquine.mp. 1993  14 Methotrexate.mp. 12891  15 Sulfasalazine.mp. 1126  16 Tumor Necrosis Factor-alpha/ai [Antagonists & Inhibitors] 0  17 (adalimumab or certolizumab pegol or etanercept or infliximab).mp. 8039  18 Antibodies, Monoclonal/ 7556  19 Golimumab.mp. 776  20 (bDMARD* or dmard*).tw,kw. 3444  21 Disease-Modifying Antirheumatic Drug*.tw,kw. 1428  22 Abatacept.mp. 968  23 Rituximab.mp. 5825  24 Antibodies, Monoclonal, Humanized/ 5976  25 Tocilizumab.mp. 1593  26 Sarilumab.mp. 321  27 Anakinra.mp. 458  28 exp Antirheumatic Agents/ 44205  29 Tofacitinib.mp. 1104  30 Baricitinib.mp. 671  31 exp Glucocorticoids/ 22293  32 Glucocorticoid*.tw,kw. 6058  33 tnf alfa inhibit*.tw,kw. 11  34 Tumor Necrosis Factor* alpha inhibit*.tw,kw. 42  35 dt.fs. or therapy.tw,kw. 704781  36 "Severity of Illness Index"/ 22722  37 (disease activity or remission).tw,kw. 47800  38 "Predictive Value of Tests"/ or "Sensitivity and Specificity"/ or predict*.tw,kw. or accurac*.tw,kw. or sensitiv*.tw,kw. 202960  39 or/12-38 872005  40 11 and 39 210  41 limit 40 to yr="2000 -Current" 205  42 limit 41 to yr="2021 -Current" 22 |
| --- |

**Supplementary Figure S1. Study Selection**


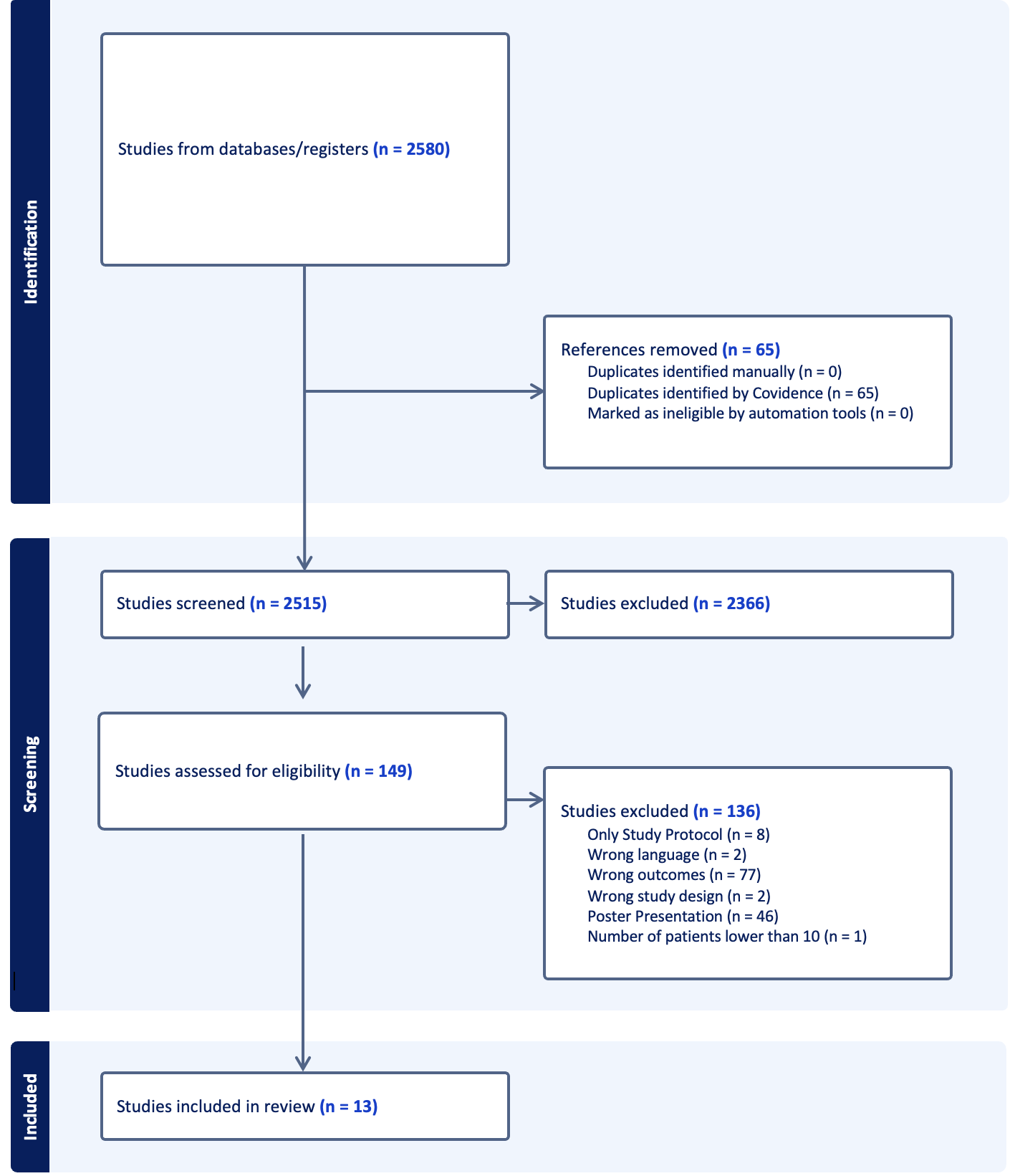


**Wrong outcomes: These studies, although presented ultrasound related information, did not provide any analysis on the prediction of Doppler, based on the baseline findings.**

**Supplementary Table S2. Outcome measures/Analysis of excluded studies during full-text review**

| **Study Title** | **First Author** | **Outcomes/Analysis related to the US findings** |
| --- | --- | --- |
| Ultrasound7 versus ultrasound12 in monitoring the response to infliximab in patients with rheumatoid arthritis | Xiaomei et al., 2016 | Significance of change in US-7 and US-12 scores as well as clinical indices, after Infliximab treatment after 6, 14, and 22 weeks. Correlations between US scores and clinical indices was also calculated at baseline and at follow up. |
| Rheumatoid and psoriatic knee synovitis: clinical, grey scale, and power Doppler ultrasound assessment of the response to etanercept | Fiocco, et al., 2005 | Significance of mean changes in clinical and US scores of rheumatoid and psoriatic refractory knee joint synovitis over 12 months follow up period |
| Ultrasound of the hand is sufficient to detect subclinical inflammation in rheumatoid arthritis remission: a post hoc longitudinal study | Hammer et al., 2017 | Percentage of patients with doppler positivity in clinical remission after 6 and 12 months of biologics treatment |
| Exploring a new ultrasound score as a clinical predictive tool in patients with rheumatoid arthritis starting abatacept: results from the APPRAISE study | D'Agostino et al., 2016 | The predictive value of changes in GLOESS at week 1–16, on clinical status and response at week 24 with abatacept. Correlations between DAS28 and GLOESS at baseline and follow up. |
| Doppler sonographic evaluation of infliximab therapy for rheumatoid arthritis | Kanno et al., 2005 | Mean change in clinical and US scores in RA patients after 3 infliximab injection |
| Contrast-enhanced ultrasonography is more sensitive than grayscale and power Doppler ultrasonography compared to MRI in therapy monitoring of rheumatoid arthritis patients | Ohrndorf et al., 2011 | Correlation of change in US scores, CRP and MRI scores after treatment in 3 and 6 months in RA patients |
| Evaluation of a new erosion score by musculoskeletal ultrasound in patients with rheumatoid arthritis: is US ready for a new erosion score? | Ohrndorf et al., 2014 | Correlation of new semicantitive erosion score (by US) with PDUS score and clinical activity indices before and after treatment. |
| Pragmatic randomised controlled trial of very early etanercept and MTX versus MTX with delayed etanercept in RA: the VEDERA trial | Emery et al., 2020 | Comparison in change of PDUS scores in 2 treatment arms in RA patients |
| Quantitative power Doppler ultrasonography is a sensitive measure of metacarpophalangeal joint synovial vascularity in rheumatoid arthritis and declines significantly following a 2-week course of oral low-dose corticosteroids | Larché et al., 2010 | PDUS score change over time after treatment and comparison of PDUS change with ESR-CRP change after treatment |
| Ultrasonographic Efficacy of Biologic and Targeted Synthetic Disease-Modifying Antirheumatic Drug Therapy in Rheumatoid Arthritis From a Multicenter Rheumatoid Arthritis Ultrasound Prospective Cohort in Japan | Nishino et al., 2018 | Correlation of baseline variables (clinical and demographic) with low PD activity or PD remission at 6 months in RA patients. |
| Clinical and ultrasound-based composite disease activity indices in rheumatoid arthritis: results from a multicenter, randomized study | Mandl et al.,2013 | Comparison the discriminant capacity between the multimodal disease activity indices (incorporating US in clinical indices) and their clinical counterparts |
| Clinical and ultrasound remission after 6 months of treat-to-target therapy in early rheumatoid arthritis: associations to future good radiographic and physical outcomes | Sundlisaeter et al., 2018 | Predictive value of clinical and US remission in 6 months on radiographic progression in 12-24 months. |
| Non-TNF inhibitor switchers versus TNF inhibitor cyclers from multicentre rheumatoid arthritis ultrasonography prospective cohort in Japan | Endo et al.,2020 | Comparison of clinical indices and US scores between TNFi switcher vs cyclers during the follow-up |
| Anti-TNF-Alpha-Adalimumab Therapy Had Time Lag of Improvement in Synovial Hypertrophy Compared to Rapid Response in Power Doppler Synovial Vascularity | Chen et al.,2017 | Significance of change in US scores and clinical remission indices after Adalimumab treatment. |
| Assessing synovitis in the hands in patients with rheumatoid arthritis by ultrasound: an agreement study exploring the most inflammatory active side from two Norwegian trials | Terslev et al.,2019 | Comparison of US scores between dominant hand vs counterpart hand in RA patients before and after treatment |
| Clinical and ultrasonographic monitoring of response to adalimumab treatment in rheumatoid arthritis | Iagnocco et al.,2008 | Evaluation of change in US, clinical scores and laboratory parameters over 2 years after Adalimumab treatment |
| High-resolution ultrasound confirms reduced synovial hyperplasia following rituximab treatment in rheumatoid arthritis | Ziswiler et al.,2009 | Assessment of the change in clinical indices and US scores change in RA patients on joint and patient level after B-cell depression by RTX treatment |
| Ultrasonographic measures of synovitis in an early phase clinical trial: a double-blind, randomised, placebo and comparator controlled phase IIa trial of GW274150 (a selective inducible nitric oxide synthase inhibitor) in rheumatoid arthritis | Seymour et al.,2012 | Synovial thickness and vascularity by US measured at day 1, Day 15 and Day 28 of oral dosing of 60 mg of the inducible nitric oxide synthase inhibitor (GW274150) or 7.5 mg prednisolone in RA patients. Change of US scores over time compared between treatment groups |
| Ultrasound of metacarpophalangeal joints is a sensitive and reliable endpoint for drug therapies in rheumatoid arthritis: results of a randomized, two-center placebo-controlled study | Seymour et al.,2012 | Comparison of change in ultrasonographic measures of synovitis in 2 weeks of 2 prednisone dose treatment groups (15mg or 7.5mg) between placebo |
| A 78-joints ultrasonographic assessment is associated with clinical assessments and is highly responsive to improvement in a longitudinal study of patients with rheumatoid arthritis starting adalimumab treatment | Hammer et al.,2010 | Correlation between change of clinical indices and ultrasound scores from baseline to 12 months |
| Longitudinal power Doppler ultrasonographic assessment of joint inflammatory activity in early rheumatoid arthritis: predictive value in disease activity and radiologic progression | Naredo et al., 2007 | The cross-sectional ad longitudinal correlations between the US parameters and the DAS28, CRP level, and HAQ score at each visit (baseline to 1 year) |
| Sirukumab and adalimumab reduce power Doppler ultrasound signal in patients with rheumatoid arthritis by 4 weeks in a phase III trial | Sweet et al.,2019 | Correlation of change between joint vascularity /thickness scores by ultrasound and disease activity score from baseline to 24 weeks after Sirukumab and Adalimumab therapy |
| Correlation between clinical activity measured by DAS-28 and ultrasound in patients with rheumatoid arthritis | Cerona et al.,2016 | Correlation was determined for the clinical, ultrasonographic and radiological scores at baseline and in 4 months after treatment |
| Clinical, Patient-Reported, and Ultrasound Outcomes from an Open-Label, 12-week Observational Study of Certolizumab Pegol in Spanish Patients with Rheumatoid Arthritis with or without Prior Anti-TNF Exposure | Blanco et al.,2020 | Significance of improvement in clinical indices, HAQ-DAI and ultrasound scores (synovial hypertrophy index, PD signal index) was assessed after CZP treatment in 12 weeks |
| Clinical, Ultrasound, and Predictability Outcomes Following Certolizumab Pegol Treatment (with Methotrexate) in Patients with Moderate-to-Severe Rheumatoid Arthritis: 52-Week Results from the CZP-SPEED Study | Sarzi-Puttini et al.,2018 | Correlation in change between DAS28 score and PDUS score over time with Certolizumab treatment in 52 week |
| Changes in Ultrasonographic Vascularity Upon Initiation of Adalimumab Combination Therapy in Rheumatoid Arthritis Patients With an Inadequate Response to Methotrexate | Kaeley et al.,2016 | Correlation between synovial vascularity score and clinical disease activity indices and disease duration at baseline. Also correlation of the change from baseline to week 24 in synovial vascularity score with the corresponding changes in DAS28-CRP or SDAI was calculated. |
| Ultrasound and magnetic resonance imaging did not provide early assessment of biotherapy response in patients with rheumatoid arthritis | Foltz et al.,2009 | Correlation of change between US, MRI vs clinical indices after treatment |
| Color flow signals of six-joint Doppler sonography correlate with disease activity in patients with rheumatoid arthritis | Kasukawa et al.,2013 | Correlation of change between color flow signals by US and DAS28 ESR-CRP after three injections of infliximab (3 mg/kg) over a 6-week period |
| Comparison of ultrasonographic assessment of synovitis and joint vascularity with radiographic evaluation in a randomized, placebo-controlled study of infliximab therapy in early rheumatoid arthritis | Taylor et al.,2004 | Correlations between synovial thickness and total van der Heijde–Sharp score in RA patients treated with infliximab and MTX |
| Monitoring anti-interleukin 6 receptor antibody treatment for rheumatoid arthritis by quantitative magnetic resonance imaging of the hand and power Doppler ultrasonography of the finger | Kamishima et al.,2011 | Correlation of baseline PDUS and MRI damage score in RA patients who are about to start IL-6 Therapy |
| Targeting ultrasound remission in early rheumatoid arthritis: the results of the TaSER study, a randomised clinical trial | Dale et al.,2016 | Comparison of change in DAS44 and core set domains in 2 treatment arms (US driven tight control arm and traditional arm) |
| Serum levels of CXCL13 are associated with ultrasonographic synovitis and predict power Doppler persistence in early rheumatoid arthritis treated with non-biological disease-modifying anti-rheumatic drugs | Bugatti et al.,2012 | Evaluation of CXCL13 levels at baseline in relation to the achievement of low disease activity (LDA, DAS < 2.4) and minimal US residual inflammation (PD ≤ 1) at 12 months after treatment |
| Application of high frequency color Doppler ultrasound in the monitoring of rheumatoid arthritis treatment | Zheng et al.,2014 | Correlation of the ESR, CRP and DAS28 score with the ultrasound parameters (synovial thickness and synovial blood flow integrity) at baseline and after TNF treatment |
| Evaluation of a novel 7-joint ultrasound score in daily rheumatologic practice: a pilot project | Backhaus et al.,2009 | Significance of change in DAS28 and PDUS scores after 6 months of treatment of cDMARD or TNF inhibitor treatment |
| Doppler sonographic evaluation of effect of treatment with infliximab (Remicade) for rheumatoid arthritis | Takahashi et al., 2005 | Correlation of baseline US with clinical change in 6 weeks of Infliximab treatment |
| High-field MRI and powerdoppler sonography: supplementary imaging, techniques in assessing disease activity in patients with psoriasis arthritis (PsA) and rheumatoid arthritis (RA) receiving adalimumab | Hoehle et al.,2013 | Significance of change in clinical activity and US scores after treatment in RA and PsA patients |
| Gray-scale and color duplex Doppler ultrasound of hand joints in the evaluation of disease activity and treatment in rheumatoid arthritis | Nvanac et al.,2015 | Significance of changes in clinical, laboratory and ultrasound parameters in 6 months after treatment (separate joint analysis) - Also correlation of change in 6 months between resistant index by US and DAS 28 scores |
| Tenosynovitis US scoring systems follow synovitis and clinical scoring systems in RA and are responsive to change after biologic therapy | Vlad et al.,2015 | Comparison of Clinical activity scores and ultrasound scores at baseline and at follow up (1st and 6th weeks) with biologics treatment |
| Radiographic prognosis of finger joint damage predicted by early alteration in synovial vascularity in patients with rheumatoid arthritis: Potential utility of power doppler sonography in clinical practice | Fukae et al.,2011 | Risk of radiographic progression according to vascularity in US at week 4 and 8 of treatment |
| Low-dose oral prednisone improves clinical and ultrasonographic remission rates in early rheumatoid arthritis: results of a 12-month open-label randomised study | Montecucco et al.,2012 | Comparison of clinical activity indices and US scores between oral low dose prednisone and MTX treatment groups in 12 months |
| Power Doppler ultrasonographic monitoring of response to anti-tumor necrosis factor therapy in patients with rheumatoid arthritis | Naredo et al.,2008 | Correlation of clinical activity indices and US scores at baseline and after TNF treatment (3,6 and 12 months) |
| Improvement of large-joint ultrasonographic synovitis is delayed in patients with newly diagnosed rheumatoid arthritis: results of a 12-month clinical and ultrasonographic follow-up study of a local cohort | Harman et al.,2015 | Correlation of radiographic damage and PDUS scores after treatment in newly diagnosed RA patients |
| Very early improvements in the wrist and hand assessed by power Doppler sonography predicting later favorable responses in tocilizumab-treated patients with rheumatoid arthritis | Kume et al.,2011 | Correlation of clinical and US scores at 2nd weeks and 24th weeks of TCZ treatment |
| Rheumatoid hand joint synovitis: gray-scale and power Doppler US quantifications following anti-tumor necrosis factor-alpha treatment: pilot study | Ribbens et al.,2003 | Significance of mean change in US scores after Infliximab treatment and correlation of change in clinical and US scores after treatment. |
| Doppler ultrasound measurements of knee joint synovitis in rheumatoid arthritis patients treated with infliximab | Kasukawa et al.,2007 | Significance of change in US scores in knee joint of 20 RA patients after 3 Infliximab infusion |
| Power Doppler ultrasound monitoring of response to anti-tumour necrosis factor alpha treatment in patients with rheumatoid arthritis | Iagnocco et al.,2015 | Correlation between the global PDUS score and DAS28 score change after TNF treatment in active RA patients in 3 months |
| The ability of synovitis to predict structural damage in rheumatoid arthritis: a comparative study between clinical examination and ultrasound | Dougados et al.,2013 | Risk (OR) of structural progression in radiography according to baseline clinical and US scores in active RA patients after TNFi treatment |
| Semi-quantitative analysis of rheumatoid finger joint synovitis using power Doppler ultrasonography: when to perform follow-up study after treatment consisting mainly of antitumor necrosis factor alpha agent | Kamishima et al.,2010 | PDUS scores were compared between ACR responders and non-responders after Infliximab treatment during the follow up for each visit (2 weeks, 6 weeks, 14 weeks, 30 weeks, 38 weeks  46 weeks, and 54 weeks). |
| Sentinel joint scoring in rheumatoid arthritis: an individualized power Doppler assessment strategy | Kuo et al.,2021 | Correlation of sentinel joint score and PDUS score in RA patients starting intravenous Tocilizumab treatment at at baseline and weeks 4, 12, 16, and 24. |
| Metrologic properties of ultrasound versus clinical evaluation of synovitis in rheumatoid arthritis: results of a multicenter, randomized study | Mandl et al.,2012 | Correlation of change during follow-up between different US scores and clinical scores were calculated RA patients in between different treatment groups. |
| Power Doppler sonography monitoring of synovial perfusion at the wrist joints in patients with rheumatoid arthritis treated with adalimumab | Filippucci et al.,2006 | Correlation of reduction in clinical and Doppler scores were calculated in clinically active RA patients after Adalimumab treatment in 2,6, and 12 weeks. |
| Validity, reproducibility, and responsiveness of a twelve-joint simplified power doppler ultrasonographic assessment of joint inflammation in rheumatoid arthritis | Naredo et al., 2008 | Correlation of change in PDUS and DAS28 scores in 3 months calculated in RA patients starting anti-TNFa therapy. |
| Positive synovial vascularity in patients with low disease activity indicates smouldering inflammation leading to joint damage in rheumatoid arthritis: time-integrated joint inflammation estimated by synovial vascularity in each finger joint | Fukae et al.,2013 | Prediction of radiographic damage has been evaluated according to PDUS at baseline and at weeks 8, 20 and 40 in RA who were administered adalimumab or tocilizumab treatment |
| Decrease in articular hypoxia and synovial blood flow at early time points following infliximab and etanercept treatment in rheumatoid arthritis | Fisher et al.,2016 | Correlation between PDUS score and arthroscopic synovitis/oxygen tension measurements, at baseline and 4 weeks after TNFi treatment |
| Tender Joint Count and Inflammatory Activity in Patients With Established Rheumatoid Arthritis: Results From a Longitudinal Study | Hammer et al.,2020 | Correlation of clinical disease activity scores and US scores was calculated and compared between RA patients groups categorized into Tender joint predominant and Swollen joint predominant. |
| Value of ultrasonography as a marker of early response to abatacept in patients with rheumatoid arthritis and an inadequate response to methotrexate: results from the APPRAISE study | D'Agostino et al.,2016 | Significance of mean change of GLOESS score and DAS28 was calculated at baseline and in 24 weeks in RA patients who received MTX and IV Abatacept treatment |
| Power Doppler ultrasound of rheumatoid synovitis: Quantification of therapeutic response | Teh et al.,2003 | Significance of mean change in doppler vascularity score was calculated active RA patients after IV prednisone treatment. |
| Predominance of large joint active synovitis in Asian patients with established rheumatoid arthritis | Cheung et al., 2016 | Correlation of US scores and HAQ score was calculated and compared in clinically stable RA patient groups with large joint and small joint involvement |
| Three-dimensional volumetric ultrasound: a valid method for blinded assessment of response to therapy in rheumatoid arthritis | Naredo et al., 2013 | Reliability of volumetric PDUS was calculated in RA patients who are about to start RTX treatment. |
| Musculoskeletal ultrasound as a biomarker of remission - results from a one-year prospective study in patients with rheumatoid arthritis | Sapundzhieva et al., 2018 | Comparison of the percentage of patients in clinical remission with persistent PD signal at 1, 3, 6 and 12 months in between 2 RA patient groups treated with sDMARD vs bDMARD. |
| Major reduction of ultrasound-detected synovitis during subcutaneous tocilizumab treatment: results from a multicentre 24 week study of patients with rheumatoid arthritis | Hammer et al.,2021 | RA patients who start sc TCZ, clinical assessments at baseline, 2, 4, 8, 12, 16, 20, and 24 weeks- US assessment baseline, 4, 12, and 24 weeks- change of the clinical and US scores calculated- and correlation of US score change and composite score changes evaluated |
| Power Doppler ultrasound assessment of rheumatoid hand synovitis | Stone et al.,2001 | Significance of mean change in US scores and clinical response after steroid treatment |
| The 6-joint ultrasonographic assessment: a valid, sensitive-to-change and feasible method for evaluating joint inflammation in RA | Perricone et al.,2012 | Correlation of changes in the DAS-28 and changes in the US scores were calculated in RA patients starting Etanercept in 3 months |
| Usefulness of power Doppler ultrasound for prediction of re-therapy with rituximab in rheumatoid arthritis: a prospective study of longstanding rheumatoid arthritis patients | Reiche et al.,2014 | US parameters and clinical activity indices were compared between patients with and without re-treated with RTX. |
| Correlation of radiographic progression with the cumulative activity of synovitis estimated by power Doppler ultrasound in rheumatoid arthritis: difference between patients treated with methotrexate and those treated with biological agents | Ikeda et al.,2013 | Correlation between total Sharp score and cumulative total PD scores and cumulative DAS28 has been analyzed in RA patients after follow up in 12 and 24 weeks of treatment |
| Ultrasound response to tofacitinib in patients with rheumatoid arthritis: Data from a multicenter 24 weeks prospective study | Germano et al.,2022 | Change in the Us score and DAS28-CRP scores during follow up after treatment. Correlation between reduction of these scores were calculated |
| Importance of baseline musculoskeletal ultrasound findings in the prognosis of rheumatoid arthritis | Sun et al., 2022 | Radiographic progression in RA patients. Association between baseline US scores and RA progression risk |
| Clinical and ultrasonographic evaluation of the window of opportunity for retreatment with rituximab in rheumatoid arthritis patients from a multicentre real-life study | Vittecoq et al., 2023 | Change in clinical /biological parameters (DAS28-ESR, CRP) and ultrasonographic scores during the 6 months of follow-up. Correlation between these changes were calculated |
| Ultrasound efficacy of targeted-synthetic disease-modifying anti-rheumatic drug treatment in rheumatoid arthritis: a multicenter prospective cohort study in Japan | Endo et al., 2022 | Change in US scores and clinical disease activity scores after 6 months follow up in between JAK inhibitor-cycler group and the interleukin-6 inhibitor inadequate response group |
| The MCP2 and the wrist plus two extensor compartments are the most affected and responsive joints/tendons out of the US7 score in patients with rheumatoid arthritis-an observational study | Podewski et al., 2022 | Change in US7 score during 3-6 follow-up. identifying the specific tendon and joints which predicts the response to treatment in RA patients |
| Association between ultrasound images and patient-reported outcomes in the treatment of rheumatoid arthritis: a retrospective study | Nawata et al., 2021 | Correlation of US scores and disease activity parameters (DAS28 CRP) at one time point |
| Predictive Role of Ultrasound Remission for Progressive Ultrasonography-Detected Structural Damage in Patients with Rheumatoid Arthritis | Li et al., 2022 | Correlation of baseline US scores with structural damage scoring findings at follow-up. |
| Joint tenderness and ultrasound inflammation in DMARD-naive patients with early rheumatoid arthritis | Sundlisater et al., 2021 | Correlation of tender joint count and PD positivity in the absence of swelling in early RA patients at one time point |
| Proliferative synovitis, an ultrasound pattern associated with ACPA-positive patients and erosive disease in rheumatoid arthritis | Garcia et al., 2022 | Association of ACPA positivity, treatment patterns and erosive changes with high doppler signal positivity in RA patients at one time point. |
| Fatigue is cross-sectionally not associated with objective assessments of inflammation, but changes in fatigue are associated with changes of disease activity assessments during biologic treatment of patients with established rheumatoid arthritis | Hammer et al., 2021 | Cross-sectional correlations between fatigue and PROMs and ultrasound scores at each visit. |
| Fatigue Is Not Associated With Objective Assessments of Inflammation During Tocilizumab Treatment of Patients With Rheumatoid Arthritis | Hammer et al., 2022 | Cross sectional and longitudinal association between fatigue and PROMs, US scores in RA patients starting Tocilizimab |
| Region specificity of rheumatoid foot symptoms associated with ultrasound-detected synovitis and joint destruction | Ishie et al., 2022 | Correlation of US-detected synovitis in the forefoot and in the midfoot with HAQ and future joint destruction. |

**Supplementary Table S3. Demographics and characteristics of studies**

| **First author** | **Inclusion Criteria** | **Baseline disease activity** | **Post-treatment disease activity** | **Change in disease activity** |
| --- | --- | --- | --- | --- |
| Gazel et al. (2023) | moderate/high disease activity according CDAI and escalation of treatment | NA | NA | The mean decrease of TJC in the 7 PD-positive patients was 7.2 ± 5.1 compared to the mean decrease in 4 PD-negative patients of 4.2 ± 2.9. |
| Ceccarelli et al. (2022) | RA patients starting treatment with baricitinib 4 mg/day or tofacitinib 5 mg/  bid due to an inadequate response or  intolerance to at least one csDMARD | Patients naive to bDMARDs treatment; DAS28CRP: median 5.0, IQR 1.8  patients previously treated  with bDMARDs:  4.2, IQR 1.9 | NR | Reduction in DAS28CRP after 4  weeks of treatment (4.85, IQR 1.76 *vs.*  3.62, IQR 1.60; *p*<0.0001) |
| Morris et al.(2021) | on stable concomitant treatment with DMARDs or no DMARDs for more than 1 month prior to enrollment, DAS28-ESR ≥ 4.4, and total power Doppler score ≥ 10 at screening | Mean DAS28-ESR= 6.4 ± 1.0 CDAI=40.5 ± 12.8 | Mean DAS28-ESR=4  CDAI= 23 | Number of patients who achieved improvement in DAS28-ESR ≥ 1.2 at 12 weeks and 24 weeks was 24/50 (48%) and 37/44 (84%) |
| Razmjou et al. (2020) | ^μ^ Baseline DAS28/ ESR ≥ 3.2, baseline total PDUS score ≥ 10, and stable ongoing treatment with a csDMARD | DAS28/ESR= 6.3 (1.2) *  CDAI (0–76) = 39.9 (13.2) * | DAS28/ESR= 4.6 (1.4) *  CDAI (0–76) = 21.6 (13.0) * | NR |
| Tanya Sapundzhieva (2018) | ^μ^ Moderate or high disease activity according to DAS28 (CRP) | **For sDMARD group=**  DAS28= 5.26 (3.42–7.34)  **For bDMARD group=**  DAS28= 5.46 (3.54–7.62) | NR | NR |
| Kawashiri (2017) | ^μ^ Starting biologic therapy for disease activity | DAS28-ESR= 5.24 (3.29–7.87)^#^ | DAS28-ESR  At 3 months=3.07  At 6 months= 2.67^#^  DAS28-ESR remission at 6 months (n)=  18 (46.2%)  EULAR good response (n)= 22 (56.4%)  EULAR moderate response (n)= 11 (28.2%)  EULAR no response (n)=6 (15.4%) | NR |
| Christensen et al. (2016) | ^β^ RA Patients that were to initiate a csDMARD or bDMARD. | **csDMARD group=**  DAS28= 4.3 (1.2) *****  **bDMARD group=**  DAS28= 4.7 (1.0) ***** | **csDMARD group=**  DAS28 remission, n (%)=21 (44.7) *****  **bDMARD group=**  DAS28 remission, n (%)=16 (29.1) ***** | **csDMARD group=**  ΔDAS28= 1.4 (1.0, 1.8)^κ^  **bDMARD group=**  ΔDAS28= 1.3 (0.9, 1.6) ^κ^ |
| Horton et al. (2016) | ^β^ New one-set RA patients who met  DAS28-CRP4v ≥2.6 at | DAS28-CRP4v: 4.9 (4.0-5.5)^#^  DAS44-CRP4v, 3.1 (2.6-3.6)^#^ | NR | NR |
| Hull et al. (2016) | ^ε^ RA with (DAS28) >5.1 on two occasions at least 1 month apart, and failed therapy with at least two DMARDs | DAS28-CRP=  5.28 ± 0.98* | DAS28-CRP=  2.36 ± 1.04* | NR |
| Inanc et al. (2016) | Seropositive, biologic-naive RA patients, about to start anti-TNF | **For Responders =**  DAS28= 5.6 ± 1.0  **For Non-responders=**  DAS28= 5.6 ± 1.4 | EULAR response (good/moderate)  64.1% | NR |
| Ranganath et al. (2015) | ^ε^ Biologic naïve, DAS28/ESR >3.2; prednisone <10 mg; total PDUS >1 for at least two MCP joints | **For PDUS <5 cohort=**  DAS28/ESR= 6.0 (0.61)  **For PDUS ≥5 cohort=**  DAS28/ESR= 6.6 (0.78) | **For PDUS <5 cohort=**  DAS28/ESR= 4.8 (1.1)  **For PDUS ≥5 cohort=**  DAS28/ESR= 3.8 (1.2) | **For PDUS <5 cohort**  ΔDAS28/ESR= 1.1 (1.21)  **For PDUS ≥5 cohort**  ΔDAS28/ESR= 2.7 (0.96) |
| Ellegaard et al. (2014) | ^ε^ Patients who remained on the same anti-TNF therapy for 12 months and had disease activity in the wrist joint on baseline ultrasound | DAS28 (CRP)= 5.1 | NR | For whole group=  ∆DAS28= -2.21 |
| Ellegaard et al (2011) | ^ε^ RA patients with wrist involevement and started anti-TNF-a | **For completers (n=78)**  DAS28= 5.14 (1.34)  **For drop-outs (n=23)**  DAS28= 5.1 (1.2) | NR | **For whole group=**  ΔDAS28= -2.16 (1.49) |
| ^δ^ The power Doppler area (PDA) is a count of the number of pixels with PDUS signal within the defined region of interest. Synovial vascularity score (Trans PDA) is the sum of all joints.  ^Ω^ The CF is the number of colour pixels divided by the total number of pixels in a region of interest (ROI).  *Final sample size which is included in the analysis are displayed.  β 1987 ACR and/or 2010 ACR/EULAR Criteria  ε 1987 ACR Criteria  μ 2010 ACR/EULAR Criteria  a MCP 2-4 (dorsal projections); wrist central, radial and ulnar projections; m. extensor carpi ulnaris tendon; elbow (posterior  projection); knee suprapatellar, lateral and medial projections; ankle central, medial and lateral projections; tarsometatarsal central, medial and lateral projections;  MTP 24; m. tibialis posterior tendon; and m. peroneus longus et brevis tendons.  b seven joints of the clinically dominant hand/foot,affected more by swelling or tenderness, using the GermanUS7-score wrist, second and third MCP and PIP, second and fifth metatarsophalangeal (MTP) joints, Palmar scan was used to assess MCP2 and MCP3 for synovitis and tenosynovitis and dorsal scan for paratenonitis. | | | | |

**Supplementary Table S4. Quality assessment of studies**

| Study | Ellegard et al., 2014 | Ellegaard et al., 2010 | | Sapundzhieva et al., 2018 | | Razmjou et al., 2020 | | Kawashiri et al., 2017 | Ranganat et al., 2015 | Inanc et al., 2016 | Hull et al., 2016 | Horton et al., 2016 | Christensen et al., 2016 | Gazel et al., 2022 | Ceccarelli et al. 2022 | Morris et al. , 2021 |
| --- | --- | --- | --- | --- | --- | --- | --- | --- | --- | --- | --- | --- | --- | --- | --- | --- |
| 1. Research question | + | + | | + | | + | | - | + | - | + | + | + | + | + | + |
| 2. Description of the study population | + | + | | + | | + | | + | + | - | + | + | + | + | + | + |
| 3. The eligible participations of study population | + | + | | + | | + | | + | + | + | + | - | + | + | + | + |
| 4. Uniform Eligibility criteria | + | + | | + | | + | | NR | + | NR | + | + | + | + | + | + |
| 5. Sample size justification | + | - | | - | | - | | - | - | - | - | - | + | - | - | - |
| 6. Exposure assessed prior to outcome measurement | NA | NA | | NA | | NA | | NA | NA | NA | NA | NA | NA | NA | NA | NA |
| 7. Sufficient timeframe to see an effect | + | + | | + | | - | | + | + | + | + | + | + | + | + | + |
| 8. Different levels of the exposure of interest | NA | NA | | NA | | NA | | NA | NA | NA | NA | NA | NA | NA | NA | NA |
| 9. Exposure measures and assessment | + | + | | + | | + | | + | + | + | + | + | + | + | + | + |
| 10. Repeated exposure assessment | NA | NA | | NA | | NA | | NA | NA | NA | NA | NA | NA | NA | NA | NA |
| 11. Outcome measures | + | + | | + | | + | | + | + | + | + | + | + | + | + | + |
| 12. Blinding of outcome assessors | + | + | | + | | + | | + | + | NR | + | + | + | NR | + | + |
| 13. Follow-up rate | + | + | | + | | NR | | NR | + | + | + | + | + | + | + | + |
| 14. Statistical analyses | + | + | | + | | + | | - | + | - | - | + | + | - | + | + |
| Total Quality Rating | Good | Good | | Good | | Fair | | Poor | Good | Poor | Fair | Fair | Good | Fair | Good | Good |
| `+`=YES  `-`= NO  NR: Not reported; NA: Not applicable: CD: Can not determine |  |  |  | |  | |  |  |  |  |  |  |  |  |  |  |
